# Radiology and Non-operative Management of Emphysematous Infections of the Pancreas, Gallbladder, and Urinary System

**DOI:** 10.7759/cureus.70955

**Published:** 2024-10-06

**Authors:** Shirvanie Persaud, Shivani T Deyalsingh, Kimberly Ramsingh, Tanzilah Barrow

**Affiliations:** 1 Surgery, Eric Williams Medical Sciences Complex, Port of Spain, TTO; 2 Radiology, Eric Williams Medical Sciences Complex, Port of Spain, TTO

**Keywords:** emphysematous cholecystitis, emphysematous infection, emphysematous pancreatitis, emphysematous pyelonephritis, interventional radiology

## Abstract

Emphysematous abdominal infections are regarded as potentially life-threatening conditions and benefit from appropriate radiological imaging for timely diagnosis and treatment planning.

A 70-year-old non-diabetic male presented with an acute abdomen and had computed tomography diagnosed emphysematous pancreatitis, cholecystitis, and pyelonephritis. Treatment included broad-spectrum antimicrobial therapy. Ultrasound-guided percutaneous drain placement into the peri-pancreatic collection permitted culture-directed antibiotic therapy and continued drainage of the purulent collection. He achieved non-operative management of the first reported case of simultaneous emphysematous infections of the pancreas, gallbladder, and urinary system. His imaging and non-operative management are described.

## Introduction

Abdominal emphysematous infections, though rare, represent a severe and life-threatening subset of bacterial infections characterized by the presence of gas within the tissues of solid organs or the walls of hollow viscera. These infections, which include emphysematous gastritis, emphysematous cholecystitis (EC), emphyse­matous pancreatitis (EP), and emphysematous pyelone­phritis (EPN), among others, present similarly to their non-emphysematous counterparts but with a significantly worse prognosis [1]. The pathogenesis is multifactorial, involving polymicrobial invasion, host factors such as immunosuppression and organ-specific predispositions like tissue ischemia or obstruction.

In this report, we present a rare case of simultaneous emphysematous infections involving the urinary system, pancreas, and gallbladder in a patient with a history of ureteric calculi. This case underscores the complex interplay between bacterial pathogens, patient conditions, and the anatomical characteristics that contribute to the development and severity of emphysematous infections. The successful non-operative management of this patient highlights the potential for targeted antibiotic therapy and percutaneous drainage in treating these typically high-mortality conditions.

## Case presentation

History

A 70-year-old hypertensive male with a history of ischemic heart disease and an ischemic cerebrovascular accident was referred to the surgical unit for three days of generalized abdominal pain, constipation, and decreased appetite. He reported a history of gallstones and a large left ureteric calculus with resultant severe hydronephrosis managed with a percutaneous nephrostomy five years prior to this admission. He had no abdominal surgeries and was a past tobacco smoker of five pack years. He was ambulatory and fully active with good social support.

On examination, he was afebrile, hypertensive, with tachycardia, and resting comfortably in no respiratory distress. The patient had generalized abdominal tenderness with guarding across the upper abdomen and left lumbar region. There was no abdominal distension and normal bowel sounds. The remaining systemic examinations were unremarkable.

Investigations

**Figure 1:**
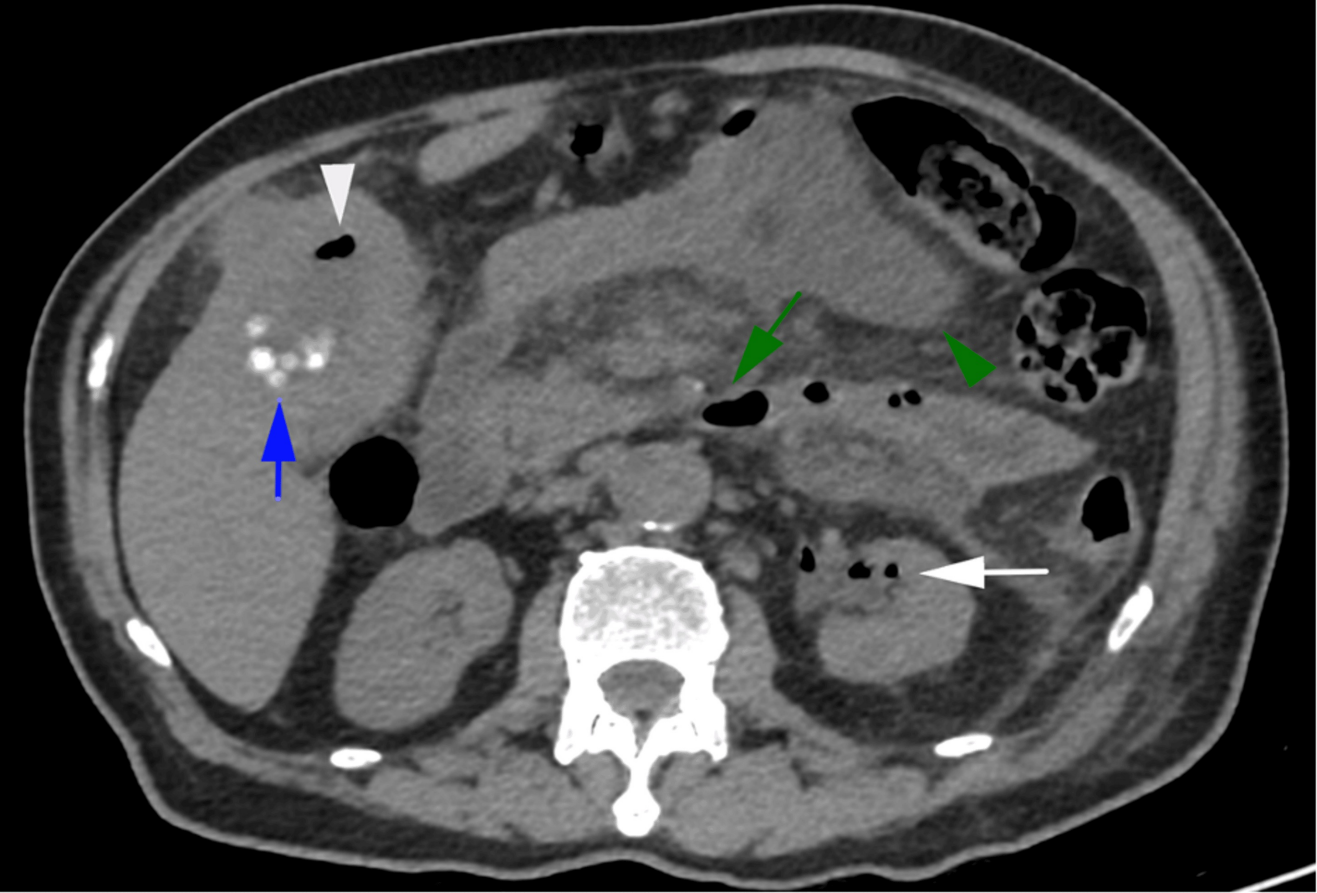
Axial non-contrast CT image of the abdomen demonstrating gas within the left kidney (white arrow), pancreas (green arrow), and gallbladder (white arrowhead) with associated cholelithiasis (blue arrow) and an early, ill-defined peripancreatic collection (green arrowhead).

**Figure 2:**
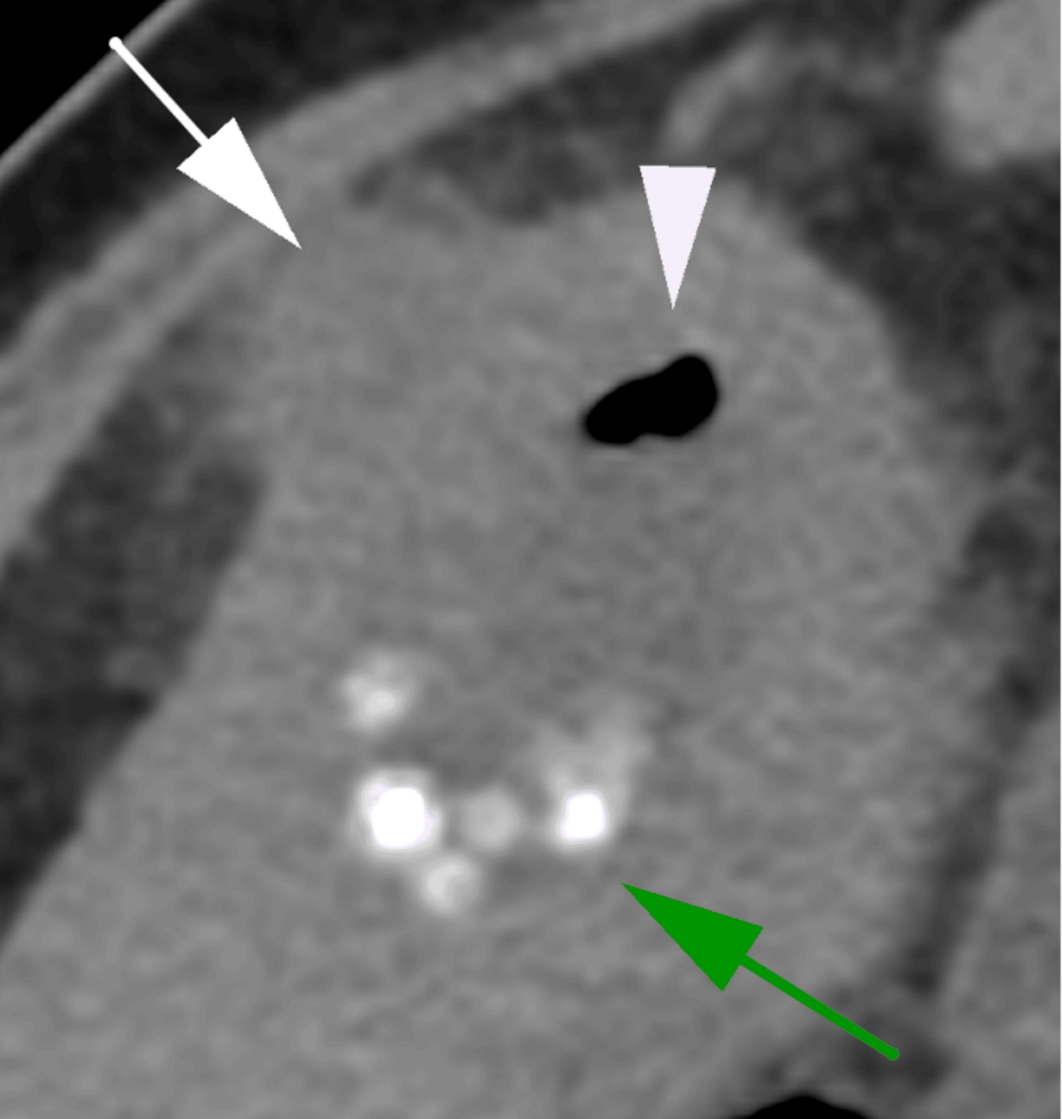
Axial non-contrast CT image of the gallbladder demonstrating gas within the gallbladder lumen (white arrowhead) with associated cholelithiasis (green arrow) and pericholecystic fat stranding (white arrow).

**Figure 3:**
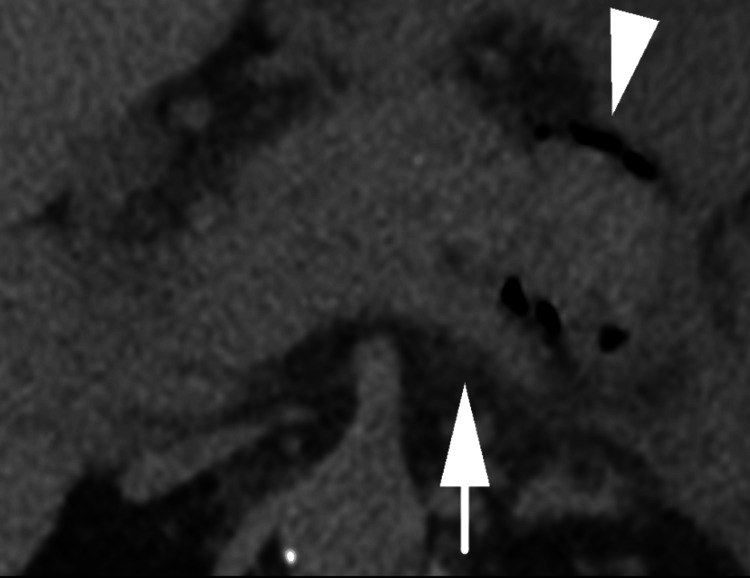
Axial non-contrast CT image of the pancreas demonstrating gas within the body of the pancreas (white arrowhead) with peripancreatic fat stranding (white arrow).

**Figure 4:**
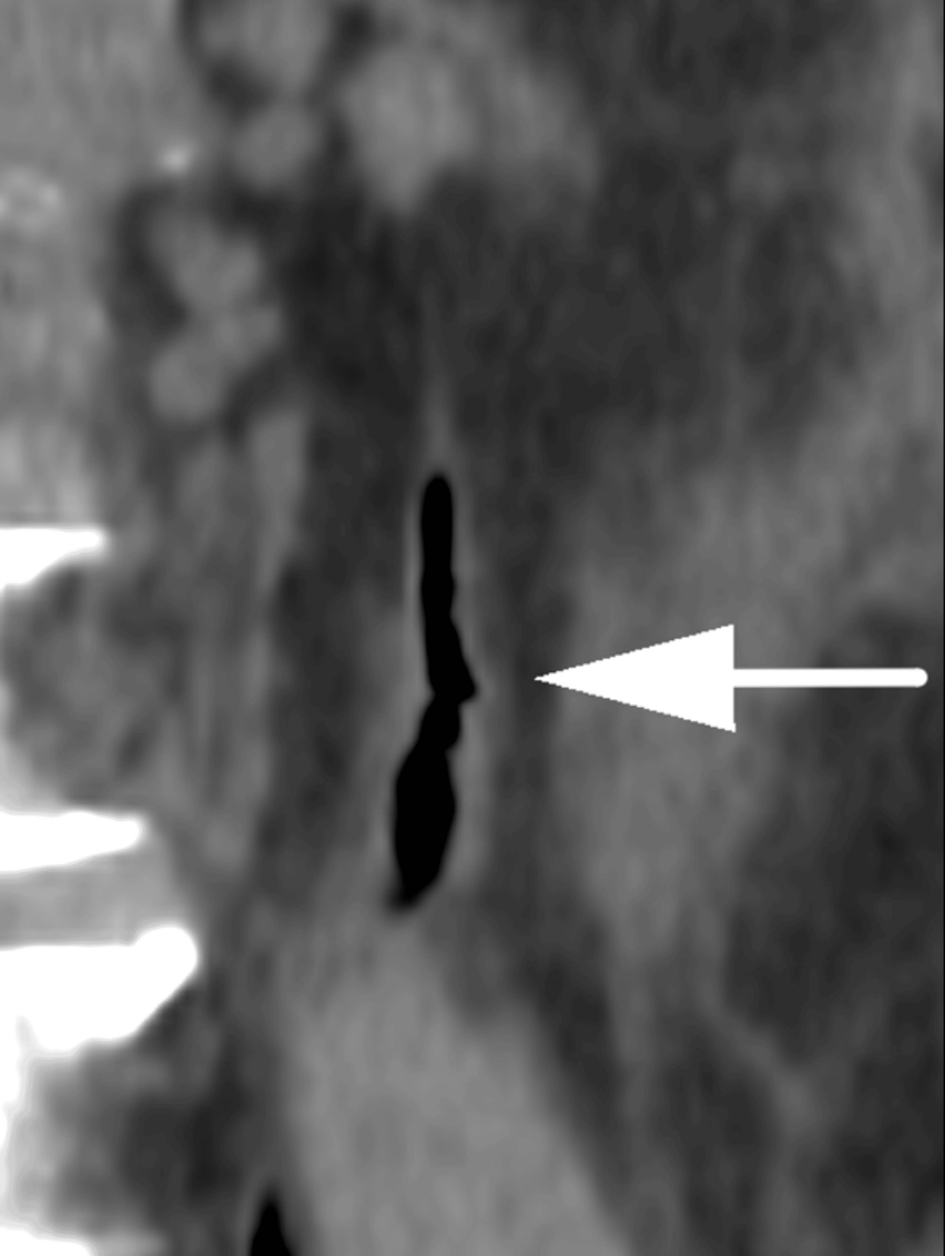
Coronal, non-contrast image of the left ureter showing gas within the ureter (white arrow).

**Figure 5:**
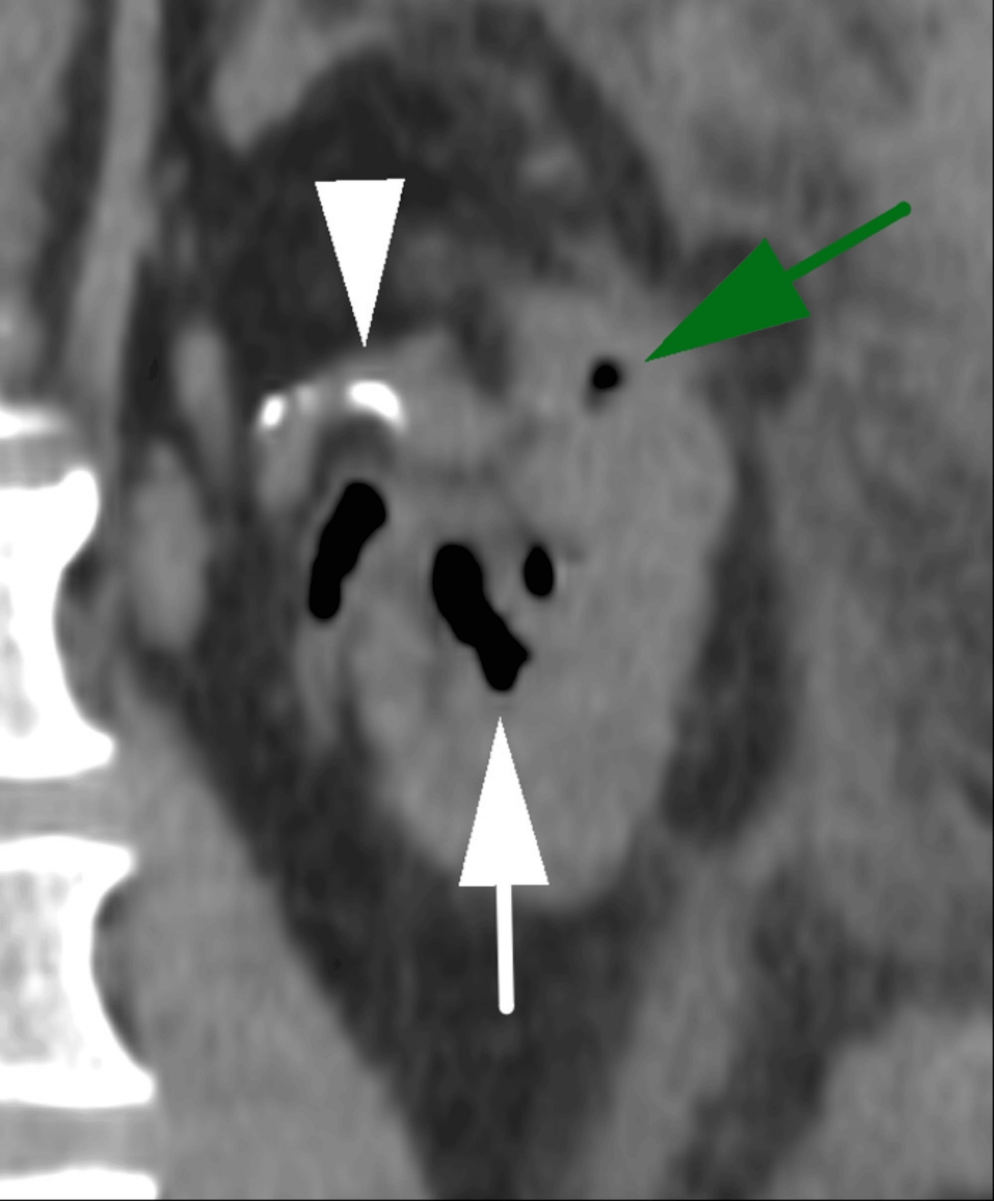
Coronal coned non-contrast image of the left kidney showing gas within the renal parenchyma (green arrow), gas within the collecting system (white arrow), and renal calculi (white arrowhead).

**Figure 6:**
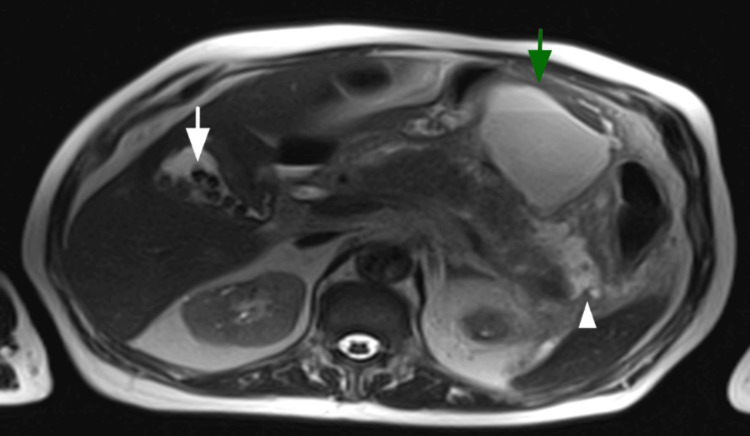
Axial MRCP showing cholelithiasis (white arrow), a peripancreatic fluid collection (green arrow), and peripancreatic free fluid (white arrowhead).

Blood investigations showed leukocytosis of 17.2 x 10^3/uL, hemoglobin of 10.8g/dL, and a platelet count of 341 x 10^3/uL. His renal function test revealed an acute kidney injury with blood urea nitrogen of 60mg/dL (normal range: 7-20) and creatinine 3.7mg/dL (0.7-1.5). His liver function tests and electrolytes were normal except for hyponatremia of 127mmol/L (137-147). Glycosylated hemoglobin (HbA1c) was 4.5mg/dL confirming his non-diabetic status. COVID antigen and PCR results were negative, and his urinalysis showed one plus blood and protein. He had blood and urine cultures taken as well as C-reactive protein. The chest and abdominal radiographs were normal. Non-contrast CT of the abdomen and pelvis (Figures 1-5) diagnosed EC, EP, left emphysematous ureteritis, and pyelonephritis. Magnetic resonance cholangiopancreatography (MRCP) showed acute cholecystitis and EP with immature gas-containing peri-pancreatic collections (Figure 6).

Treatment

**Figure 7:**
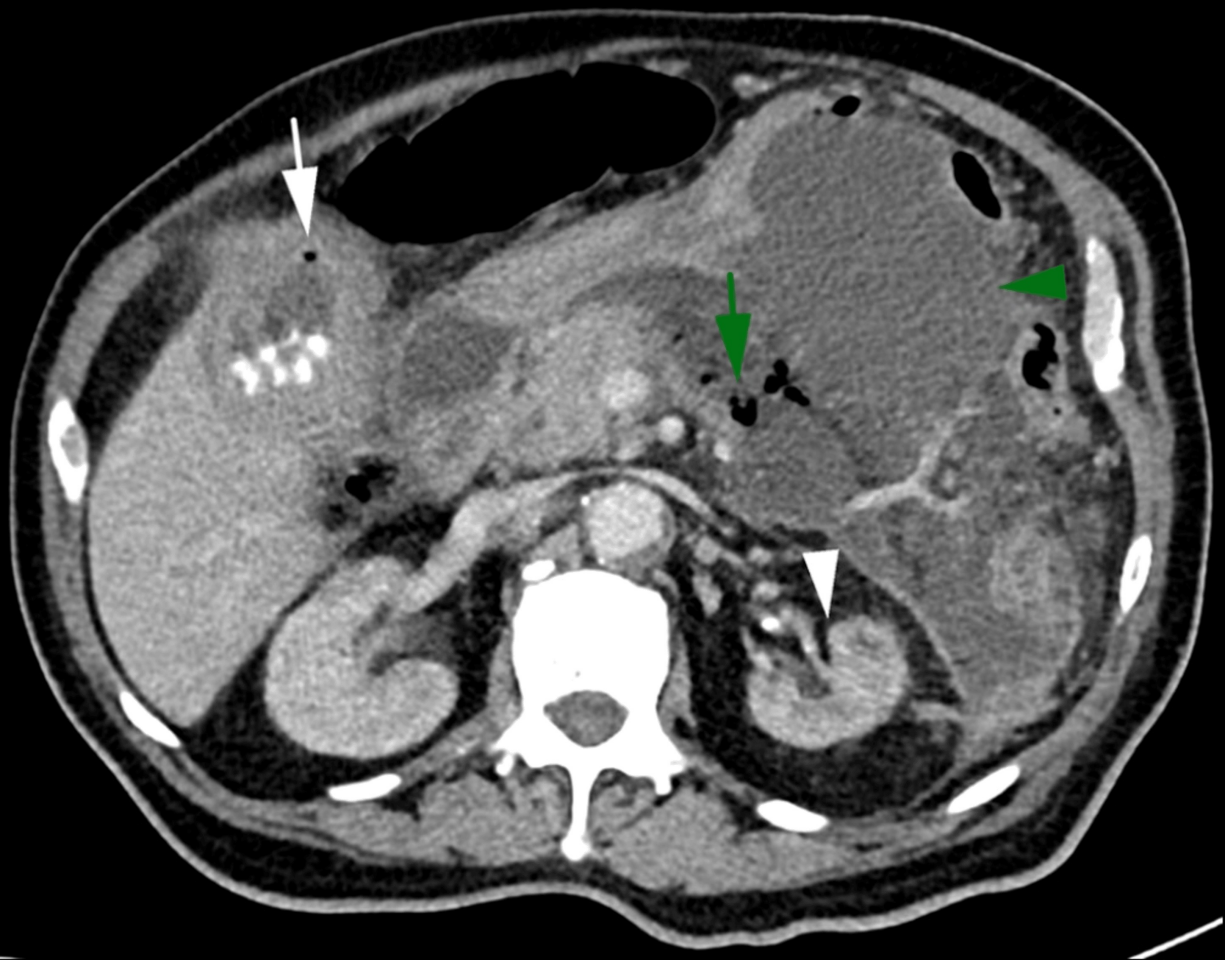
Axial contrast enhanced CT of the abdomen in the portal venous phase performed five days later demonstrating a reduction in the volume of gas within the gallbladder (white arrow), a reduction in the gas within the left kidney (white arrowhead), unchanged pancreatic gas (green arrow), and a larger, more defined peripancreatic collection (green arrowhead).

**Figure 8:**
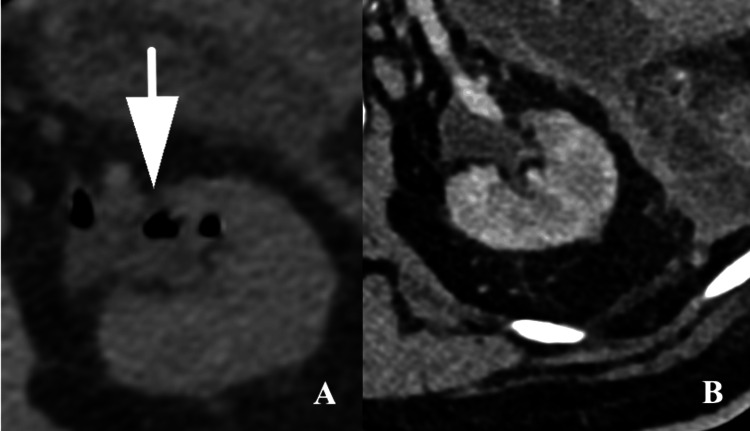
A) Axial non-contrast CT of the left kidney showing gas within the renal collecting system on the initial imaging, and B) Axial contrast enhanced CT of the left kidney five days later showing near complete resolution of this gas.

Multi-disciplinary team (MDT) management included consultations from urology, general surgery, infectious disease, nephrology, and radiology. He was treated initially with intravenous meropenem and ciprofloxacin antibiotics and intravenous fluid resuscitation with resolution of the leukocytosis and acute kidney injury. A five-day interval CT scan of the abdomen and pelvis, with intravenous contrast, showed resolution of the EPN; a reduction of the gas within the gallbladder lumen; a more defined peripancreatic collection measuring 7.5cm CC x 7.4cm AP x 6.2cm TS; and moderate intra-abdominal free fluid (Figures 7, 8). The peripancreatic collection was percutaneously drained under ultrasound guidance. The output appeared purulent and turbid, and samples were sent for Gram stain, culture, and sensitivity. His blood and urine cultures showed no bacterial growth, while his peri-pancreatic fluid cultured *Escherichia coli* and *Klebsiella pneumoniae* (Table 1). Sensitivity results allowed the prescription of oral amoxicillin-clavulanic acid, and he was discharged for weekly reviews of his drain output in the outpatient clinic.

**Table 1:** Culture and antibiotic susceptibility of peripancreatic fluid collection.

1+ *Escherichia coli*	
Sensitive to:	Resistant to:
Amoxicillin-clavulanic acid, Ceftriaxone, Piperacillin-Tazobactam, Tobramycin	Ampicillin, Metronidazole
1+ *Klebsiella pneumoniae*	
Sensitive to:	Resistant to:
Amoxicillin-clavulanic acid, Ceftriaxone, Piperacillin-Tazobactam, Tobramycin, Levofloxacin	Ampicillin, Metronidazole

Outcome and follow-up

There was a gradual decrease in drainage, permitting drain removal on the seventh week after insertion.

The patient was counseled for an elective left nephrectomy but refused surgery.

## Discussion

Abdominal emphysematous infections are characterized by the presence of gas within the parenchyma of solid organs or the walls of hollow viscera and include emphysematous gastritis, EC, EP, EPN, emphysematous cystitis, Fournier’s gangrene, and gas gangrene of the uterus. These tend to present similar to their non-emphysematous equivalent and may be caused by a variety of benign or pathological entities. Benign etiology includes the introduction of atmospheric air at instrumentation or recent surgery or by reflux from an adjacent hollow viscus, tissue infarction, or fistula formation [1].

The pathogenesis of emphysematous infections relies on the interactions of polymicrobial infections, along with patient factors and organ-specific factors, to predispose to infections and encourage bacterial propagation [2]. Small vessel disease and the presence of arteriosclerosis perpetuate tissue ischemia [2]. Defective cell-mediated immune response and tissue necrosis encourage bacterial propagation and increased gas production [2]. Elevated tissue glucose levels in patients with diabetes mellitus aid in carbon dioxide production from fermentation [3]. Hydrogen, or nitrogen gas, is also produced [2]. Thrombosis of small subcutaneous vessels ensues, with rapid gangrene of the surrounding skin and underlying fascia [1,2]. Organ-specific factors that optimize bacterial growth include bile stasis or alterations of bile secretion and its bactericidal pH, obstruction to the urinary system, and ischemia seen in gynecologic neoplasms [1].

EPN, regarded as a fulminant type of necrotizing pyelonephritis, has gas within the renal parenchyma and perirenal space [4,5]. *E. coli *or *K. pneumoniae* are the bacteria isolated in 65% to 100% of cases of urological emphysematous infections [6]. This patient had both *E. coli *and* K. pneumoniae* cultured from percutaneously drained abdominal fluid, supporting a possible urological origin of this infection. His history of ureteric calculi and previous obstruction showed a predisposition to urinary infection. Other causes of pyelonephritis are *Proteus*, *Enterococcus*, *Pseudomonas*, *Clostridium*, and rarely, *Candida *spp., and *Aspergillus *[5-7]. Two types of EPN are described based on radiological imaging: type I is characterized by parenchymal destruction, and type II has either renal or perirenal fluid collection, along with gas in the collecting system. This distinction was important because the index patient was shown to have type II EPN, which is associated with lower mortality and a better prognosis [4,5]. Emphysematous pyelitis describes gas-forming urinary tract infections that are associated with gas, localized only in the renal collecting system [8]. On plain radiographs, this can appear as gas outlining the pelvi-caliceal system [8]. There may be confusion between renal gas and gas from the overlying bowel. Ultrasonography (US) is therefore recommended in immunocompromised patients with urinary tract infections. High-amplitude, flat anterior margin echoes within the renal sinus or calyces, associated with distal shadowing containing low-level echoes and reverberations, portray the emphysematous urinary infection. This “dirty shadowing” is differentiated from the "clean shadowing" that occurs distal to renal calculi with no echoes. US provides less specific information about emphysematous urinary infections because of the difficulty of distinguishing between shadowing from air versus renal calculi or calcifications within the kidney [5,8]. Therefore, CT is the more reliable imaging technique. Ureteric stones are precisely defined and differentiated from pelvic-caliceal or perinephric gas collections [8]. CT is also useful to determine the extent of gas within the renal parenchyma, collecting system, and perinephric system and is therefore utilized for classification [9]. Emphysematous pyelitis is treated with antimicrobial therapy and relief of any urinary tract obstruction. Patients with involvement of the kidneys or extrarenal space benefit from percutaneous drainage of gas and purulent material. In the absence of obstruction, percutaneous or surgical procedures are rarely needed, and EPN likely responds to intravenous antimicrobial therapy alone [8]. Nephrectomy is reserved for extreme patients who have no improvement or who deteriorate despite optimal antibiotics and drainage.

EP is a severe type of acute necrotizing inflammation of the pancreas characterized by gas within or around the pancreas. It is caused by the above-mentioned polymicrobial infections that originate from the bloodstream or lymphatics, translocate from the adjacent colon, or reflux through the ampulla of Vater [1,10]. EP in this patient's case could have been contiguous spread from the EPN. EP is usually seen in debilitated patients with uncontrolled diabetes or chronic renal failure [11]. Radiography and sonography have limited sensitivity and specificity in localizing gas in the pancreatic bed. This is compounded by any associated ileus and overlying bowel gas [11]. CT is again the modality of choice to characterize pancreatic and peripancreatic gas [11]. CT can also reveal pancreatic necrosis, abscesses, and vascular thrombosis or pseudoaneurysm formation [10,11]. EP is treated with broad-spectrum antibiotics, and aggressive surgical intervention may be necessary. Image-guided percutaneous drainage of collections is a recommended alternative [12]. Reports of successful management of EP with conservative treatment have been published [12-14]. Early treatment avoids the high mortality rate of EP [11].

EC is not usually associated with cholelithiasis but rather caused by secondary infection from *E. coli*, *Staphylococci*, *Streptococci*, *Pseudomonas*, *Klebsiella*, and *Clostridium welchii* [15, 16]. Cases of EC are usually associated with critically ill patients in the ICU, with sepsis and systemic hypoperfusion [17]. The typically affected population is males in their fifth to seventh decade and commonly diabetic [15,18]. Peritonism is usually absent, but crepitus in the adjacent abdominal wall may rarely be palpated. Mild to moderate unconjugated hyperbilirubinemia may be caused by bacterial hemolysis. EC is often associated with gangrene, perforation, and other local and systemic complications [15,18]. Abdominal radiography may detect gas within the gallbladder fossa but is poor at differentiating EC from other causes of gas in this location [1]. Three radiographic stages of EC have been described: stage I is gas confined to the lumen; stage II has gas within the gallbladder wall, and stage III has gas extending into the pericholecystic region [11]. In the US, the air foci appear echogenic with dirty posterior shadowing due to reverberation artifacts [11]. A large amount of gas is seen as a wide band of acoustic shadow [11,19,20]. Sonographically, it may be difficult to differentiate EC from shadowing from cholelithiasis in a contracted gallbladder or calcification in a porcelain gallbladder [1,11]. CT diagnoses EC definitively and can show features of inflammation and exclude other differential diagnoses. Complications, namely abscess formation and gallbladder perforation, are also revealed [11]. Emergency cholecystectomy is the surgical treatment of EC. Critically ill patients, who are unable to tolerate the general anesthesia, benefit from percutaneous cholecystostomy. EC has a higher mortality rate of 15%, compared with a rate of less than 4% in uncomplicated acute cholecystitis [16].

Emphysematous infections portend a grave prognosis, much worse when seen in more than one organ system. Reports of combined emphysematous infections are rare but have been reported simultaneously in the gallbladder and pancreas [17]. This case report involved simultaneous emphysematous infections of the urinary system, pancreas, and gallbladder. The patient’s only predisposing factor was a history of ureteric calculi that was passed five years prior to presentation. Cholelithiasis can predispose to cholecystitis and pancreatitis but is not usually associated with emphysematous infections. MRCP showed no sign of gallstone pancreatitis or choledocholithiasis. The patient had percutaneous drain placement into a (peri)pancreatic collection. This allowed culture and sensitivity to target antibiotic therapy and continued drainage of purulent fluid. The drain was left in place until minimal output was noted. This case was resolved by successful non-operative treatment of emphysematous abdominal infections.

## Conclusions

Emphysematous abdominal infections involving the gallbladder, pancreas, stomach, or genitourinary system benefit from appropriate radiologic imaging for timely diagnosis and treatment planning. Treatment includes correction of associated acid-base and electrolyte imbalances, hypovolemia, and hyperglycemia, along with broad-spectrum antimicrobial therapy.

A 70-year-old, non-diabetic male had CT-diagnosed type II EPN, pancreatitis, and cholecystitis. He developed a peripancreatic collection that was drained percutaneously and cultured to direct antibiotic therapy. The result was non-operative management of emphysematous abdominal infections of the three organ systems.
